# The Pre-Dialysis Care Trajectory of Chronic Kidney Disease Patients and the Start of Dialysis in Emergency: A Mixed Method Study Protocol

**DOI:** 10.3390/ijerph16245010

**Published:** 2019-12-09

**Authors:** Maxime Raffray, Sahar Bayat, Arnaud Campéon, Laëtitia Laude, Cécile Vigneau

**Affiliations:** 1 Univ Rennes, French School of Public Health (EHESP), Pharmaco-epidemiology and health Services Research, (REPERES)-EA 7449, 35043 Rennes, France; sahar.bayat-makoei@ehesp.fr; 2 EHESP, Arènes, CNRS, UMR 6051, 35043 Rennes, France; arnaud.campeon@ehesp.fr; 3 EHESP, Health Organizations Management (MOS)-EA 7348, 35043 Rennes, France; laetitia.laude@ehesp.fr; 4 Department of Nephrology, Centre Hospitalier Universitaire, 35000 Rennes, France; cecile.vigneau@chu-rennes.fr; 5 Univ Rennes, Inserm, UMR 1085, 35000 Rennes, France

**Keywords:** chronic kidney disease, care trajectory, mixed methods, emergency start dialysis, big data

## Abstract

Chronic Kidney Disease (CKD) is an important public health issue that requires early and close medical monitoring to start Renal Replacement Therapy (RRT) in the best conditions. However, in France, about 1/3 of patients start dialysis in emergency, despite the existence of CKD management guidelines. Using both quantitative and qualitative methods, we wanted to analyze the pre-dialysis care trajectory of patients with CKD and document the causes of Emergency dialysis Start (ES). To this aim, we designed a convergent mixed-method study. The quantitative component will analyze individual healthcare consumption and clinical data to identify the risk factors of ES by comparing the trajectories of patients who started dialysis in emergency in 2015 in France with those of patients who started in a planned manner and with the national recommendations. The qualitative component will explore the patients’ trajectories and identify barriers to a planned start using semi-structured interviews with patients who started dialysis in emergency and with their general practitioners and nephrologists. Using the strengths of a mixed methodology, this study will bring robust and valuable findings to improve the care of CKD patients.

## 1. Introduction

Worldwide, Chronic Kidney Disease (CKD) and particularly, End-Stage Renal Disease (ESRD), its last stage of progression, represent a major public health issue. The number of people receiving Renal Replacement Therapy (RRT) was over 2.6 million in 2010 [1] and has been rising during the last decade. Recent projections suggest the same trend for the coming years [2,3].

In order to delay the progression of CKD and ensure that the patient starts RRT in the best conditions, the coordinated work of the General Practitioners (GP) and nephrologists is key [4,5]. This work includes the screening and diagnosis of CKD, the monitoring of the disease(s), the referral to a nephrologist, as well as the patient therapeutic education and preparation for RRT.

Clinical practice guidelines on the care of non-dialyzed patients with CKD have been developed and published [6], including in France [7]. However, according to the French Renal Epidemiology and Information Network (REIN) registry, each year, around 30% of incident patients with ESRD still start dialysis in emergency in France [8]. This proportion did not decrease since the publication of the CKD care guidelines in 2012. Emergency dialysis Start (ES) in REIN is defined as a first dialysis session initiated during the 24 hours following a nephrologist’s evaluation due to a life-threatening risk for the patient [9]. This definition does not exclude acute decompensation, despite an early and regular follow-up.

Such a start has negative consequences for the patients and their care trajectory. Indeed, ES is associated with higher morbidity and mortality risks [10,11,12,13,14,15] and with lower quality of life [16]. However, some evidence suggests that a longer pre-dialysis care is not associated with a lower mortality [17]. A lower chance of access to the renal transplantation waiting list also has been reported for patients who start dialysis in emergency [12]. Moreover, it has been reported that ES patients are more comorbid than patients who start dialysis in a planned manner (PS) [15,18]. Not surprisingly, late referral to a nephrologist has been associated with ES [19,20]. 

However, little is known about the care trajectories leading to late referral and ES. 

Our overarching objective is to document the causes of ES by studying the pre-dialysis care trajectory of these patients using a mixed method design. This article describes the study design and methodology to show the value and strengths as well as the challenges of such approach that could be widely used for public health research, particularly for the study of chronic diseases. 

## 2. Materials and Methods

### 2.1. The Mixed Method Design and Its Rationale

Qualitative and quantitative methods can be combined in many different ways [21]. For the aim of this research (i.e., to identify the causes of ES by studying the pre-dialysis care trajectories), a convergent design was chosen in which quantitative and qualitative data are collected and analyzed separately before merging the results and discussing the extent to which they create a better understanding of the issue [21].

Such a convergent design was selected with the aim of bringing together the strengths of both quantitative and qualitative approaches for the study of the care trajectories of patients with a chronic disease, which we consider as our central research object. The driving principle is to collect data that are different in nature but complementary in order to better understand the underlying problem [22]: in our case, the pre-dialysis care trajectory leading to an emergency dialysis start. The quantitative study brings trends and generalizations using a large sample, while the qualitative study adds depth and details using a smaller sample [23].

**Figure 1:**
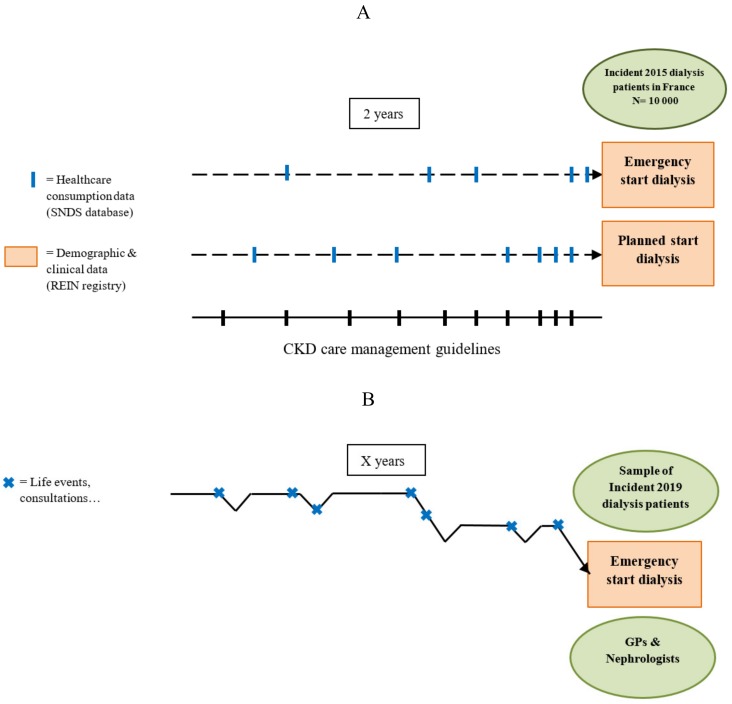
The pre-dialysis care trajectory studied using the components of the mixed method: (**A**) The quantitative component, and (**B**) The qualitative component. SNDS, French national administrative healthcare database; REIN, Epidemiology and Information Network; GP, general practitioner.

This convergent design appears to be particularly suitable for research on the care trajectories of a chronic disease and on the inadequate use of or access to healthcare services. Figure 1 illustrates how the same object of research (i.e., the pre-dialysis care trajectory) is seen differently, depending on the nature of the study component.

Studying the association between ES and quantitatively measured factors, such as the frequency of consultations with GPs and nephrologists, the prevalence of comorbidities and other clinical factors, is essential. However, the care trajectory cannot be defined only by these factors. The ways patients view and manage their disease(s), the actions taken (or not taken) and the underlying motivations, but also to a larger extent, their life experiences, are as essential in the definition of the care trajectory and must be investigated in a complementary manner. Moreover, the care trajectory is heavily influenced by the individual practices of the involved health professionals, and in our case, by the coordinated work of GPs and nephrologists [24]. One example might be difficulties to obtain a consultation with (or be referred to) a nephrologist in a reasonable interval of time. This emphasizes the need for studying the pre-dialysis care trajectory through two different lenses. 

### 2.2. The Quantitative Study

In the quantitative component of this research, the pre-dialysis care trajectory is studied as a succession of healthcare consumption events in a large number of patients across time (Figure 1A). 

#### 2.2.1. Hypotheses

We hypothesize that the pre-dialysis care trajectory of ES patients is different from the national guidelines for CKD care management (Table 1) and from the pre-dialysis care trajectory of PS patients. Specifically, we hypothesize that ES patients do not receive the minimal CKD follow-up recommended by the national guidelines. Moreover, we postulate that the frequency of consultations with GPs and nephrologists and of laboratory monitoring is lower for ES patients than for PS patients during the 2 years before dialysis start. Consequently, patients are less prepared for dialysis.

#### 2.2.2. Objectives

The objectives of this quantitative component are: (1) to compare the pre-dialysis care trajectory of ES patients with the trajectory recommended by the national guidelines; (2) to compare the pre-dialysis care trajectory between ES and PS patients; (3) to identify factors associated with ES; and (4) to identify groups of patients with similar pre-dialysis care trajectories (i.e., types of trajectories).

#### 2.2.3. Study Population

The quantitative study included all adult patients who started dialysis in France in 2015 (incident patients), extracted from the REIN registry. This registry records all patients with ESRD treated by dialysis or renal transplant in France (mainland and overseas) [25]. Each year, nearly 10,000 patients with CKD begin dialysis and are registered in the REIN registry. Patients with unknown dialysis start status (ES or PS) were excluded. 

#### 2.2.4. Data Collection

The REIN registry includes the following patient data: age, sex, residence postcode, clinical characteristics at RTT initiation (primary renal disease, serum albumin, and hemoglobin), comorbidities (diabetes, cardiovascular diseases, cancer, chronic respiratory disease, mobility) and modalities of ESRD management (vascular access, hemodialysis or peritoneal dialysis). However, it does not contain information on the pre-dialysis care trajectory. Therefore, the REIN registry was linked to the French national healthcare database (SNDS). This administrative database covers almost 99% of the population and collects data on ambulatory care (consultations, laboratory tests, drug prescriptions) and hospital stays (duration, diagnostic codes, procedures) [26]. 

The linkage of these two database types (clinical and administrative data with healthcare utilization) will give a valuable tool for public health research, especially for the study of chronic and multifactorial diseases, such as CKD. Indeed, it will increase the quantity of information available at the individual level, enabling a more comprehensive understanding of the risk factors and outcomes [27,28,29,30,31]. An iterative and deterministic approach will be used to link the 2015 incident patients included in the REIN registry with the SNDS database, using the following indirect identifiers: patient’s sex, age, residence postcode, RRT center identification number, month and year of dialysis start, month and year of death.

In the framework of our quantitative component, this linkage will allow us to retrieve and piece back together the pre-dialysis care trajectory of each patient, along with their demographic and clinical characteristics. The French national guidelines recommend starting the patient’s preparation for RRT (arteriovenous fistula, catheter for peritoneal dialysis procedures and other hospitalizations related to preparatory care for dialysis) at least 1 year before its foreseeable occurrence (Table 1). Therefore, the study period covered the 2 years before dialysis start.

**Figure 2:**
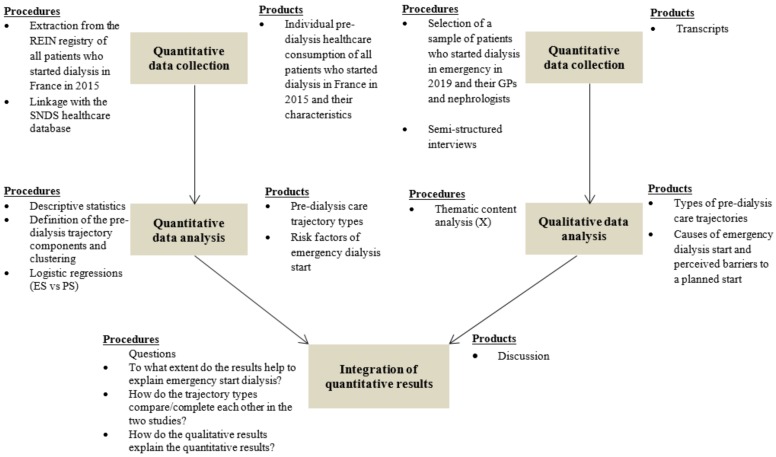
Diagram of the convergent mixed method design.

This trajectory, seen through the healthcare consumption lens, included the follow-up items recommended by the national clinical guidelines: consultations with GP and nephrologist, and laboratory tests (e.g., creatinine, potassium, calcium measurement) (Table 1). The quantitative component does not investigate what happens beyond the healthcare consumption (Figure 2). This quantitative study was approved by the French Data Protection Authority (Commission Nationale Informatique et Liberté; n 917021). 

#### 2.2.5. Analysis

After the description of the baseline characteristics (i.e., at dialysis initiation), the items of care specified by the national guidelines, retrieved for all patients, were compared with their recommended frequencies (e.g., what percentage of patients saw the nephrologist at least once every 3 months during the year before dialysis start?) (Table 1). The key components of the pre-dialysis care trajectory were defined: number and frequency of consultations with GPs and nephrologists, frequency of laboratory tests (regular, irregular or absent), frequency of hospital stays, and presence of dialysis preparation procedures. These variables were then compared between groups (ES vs. PS) using the Chi-square and t-tests. The factors associated with ES were assessed using logistic regression models that were adjusted for several characteristics (age, sex, comorbidities, primary renal disease, and pre-dialysis healthcare consumption). Finally, groups of patients who share similar care trajectories, based on the identified healthcare consumption variables, were determined using Multiple Correspondence Analysis and Hierarchical Cluster Analysis methods.

### 2.3. The Qualitative Study 

In the qualitative component of this research, the pre-dialysis care trajectory is defined as the combination of the views, actions and experiences of a restricted number of patients, the views and practices of their main health professionals involved in the care management of CKD (GPs and nephrologists), as well as the product of the interactions of all these actors (Figure 1B).

#### 2.3.1. Hypotheses

Here, we hypothesize that the causes of ES are multifactorial and linked to the patients, the health professionals, and the healthcare organization. Specifically, we hypothesize that due to CKD silent and progressive nature and due to the perceived burdensome nature of dialysis as a treatment, ES patients might show reluctance and try to delay dialysis start. We also hypothesize that GPs and nephrologists might not know exactly the precise role in CKD management of each other, thus impairing the collaborative work required for a planned start. Finally, we postulate that the national CKD care management guidelines are insufficiently known and implemented by GPs and nephrologists. 

#### 2.3.2. Objectives

The objectives of the qualitative component are: (1) to identify to what extent GPs and nephrologists know and implement the national CKD care management guidelines; (2) to describe the GPs and nephrologists’ practice regarding CKD management; (3) to reconstruct the patients’ trajectories through their views and experience and to identify the barriers to a planned dialysis start; (4) to identify types of pre-dialysis care trajectories of ES patients.

#### 2.3.3. Underlying Theory

The sociological meaning of being ill and its implications have changed during the last century, mainly due to the epidemiological transition from acute infectious diseases to non-communicable chronic diseases. Now, diseases are no longer just a parenthesis in the patient’s life and the recovery, or healing, as an ending point, became a necessary daily management of the disease [32]. Corbin and Strauss developed the pioneer concept of “illness trajectory”, offering a sociological framework to study the trajectory of patients with chronic diseases. In this concept, the trajectory is “not only the physiological development of the disease but also the work involved in its management, the impact of illness, and the changes in the lives of the ill and their families that in turn affect the management of the illness itself” [33]. This trajectory is shaped by the illness nature, the patient’s response to it, and the actions undertaken by the patient and health professionals to manage the illness. For analytical purposes, several phases shape the trajectory with turning points, critical junctures between them: pre-diagnosis, diagnosis, acute, comeback, stable, unstable and downward. Not all of these phases are present in one trajectory, whereas some might be observed several times in another. The concept of “illness trajectory” is at the core of this qualitative study, in the construction of the interview guides and in the choice to focus on the critical junctures of the patient trajectory and the GPs and nephrologists’ practices. The analytical tool of trajectory phasing was also used to describe types of trajectories of patients starting dialysis in emergency. 

#### 2.3.4. Study Population

A previous spatial analysis in the Bretagne region of France found that the western and eastern parts of this region have higher and lower risk of ES, respectively [34]. These geographical areas will be our investigation field. All adult patients with ESRD who will start dialysis in emergency and live in and around these areas in 2019 will be recruited. Patients will be identified using the REIN registry inclusion data. On the basis of the available REIN data, we expect to include about 40 patients (20 for each area). However, recruitment will be stopped upon reaching data saturation (i.e., when no new information will arise from the interviews) [35]. Beforehand, the study will be presented to the medical teams of the dialysis centers where the included patients are treated. The medical teams/staff will then seek the patients’ consent, using an information notice, and will schedule an appointment. Patients will be interviewed in-person during a dialysis session. The interviewer will ask again for their consent before starting the interview. During the interview, patients will be asked to give the name of their GP(s) and nephrologist(s) who will be interviewed in the second part of this study. To favor consistency, the same interviewer will conduct all interviews. This qualitative study is compliant with the methodological frameworks defined by the French Data Protection Authority and has been registered by the French National Institute of Health Data (n 0609030619).

#### 2.3.5. Data Collection

To reconstruct the trajectories of ES patients and how their illness was managed by themselves and their GPs and nephrologists, three interview guides (one for each actor) will be used (available in Supplementary Materials). For patients, the interview will start with few basic socio-demographic questions. The interview guide was developed to focus on and around the critical junctures that shape the trajectories and to address (1) the first signs (How did you learn that you were suffering from CKD...); (2) the medical follow-up (What do you remember of your visits to the GP/nephrologist…); (3) the adjustment to the disease and CKD strategic management (Before dialysis, how did your life change with CKD…); and (4) the process of transition to dialysis (How did your first dialysis go? What did you know about it before?…). For the health professionals, the interview will explore (1) the everyday practice regarding CKD (As a GP, what do you think your role is towards patients with CKD? What does CKD co-management mean to you?…); (2) their knowledge and implementation of the CKD care management guidelines (What do you think about these guidelines…); and (3) the process of transition from CKD to ESRD (How is discussed the need to start dialysis with a patient? Have you ever had patients who started dialysis in emergency, and if so what happened?...).

The interview guides were discussed with GPs and nephrologists and tested with patients and health professionals in real conditions. Appropriate adjustments were made (mainly rephrasing) and additional tests were performed to ensure that the data collected on the trajectory were precise enough for analysis. The interviews will be audio-recorded (after consent) and transcribed. 

#### 2.3.6. Analysis

First, a thematic analysis will be carried out to analyze the interview transcripts [36]. In this method, the researcher first becomes immersed in the data by reading the full transcripts and writing down notes to grasp the overall sense of the interviews and the information they bring. Transcripts are then read again, word by words, and data are associated with as many categories as necessary to describe all aspects of the content. This process of category generation is called “open coding”. Similar categories are progressively merged to produce a final list of categories. Coding and category generation will be performed by two different researchers to increase coherence and validity. 

The results of the thematic analysis will provide a preliminary understanding of the pre-dialysis care trajectories of ES patients. The second step will consist in identifying what makes patient trajectories similar or different from each other, and in retrieving the “illness trajectory” phases in order to describe types of trajectory leading to an emergency dialysis start. Concomitantly, a typology of the GPs and nephrologists’ practices will be performed to understand their attitudes towards the CKD care management guidelines and to what extent and how their practices shape the patients’ trajectories. Finally, the causes of an emergency dialysis start and the barriers to a planned start will be summarized.

### 2.4. Integration of Quantitative and Qualitative Results

The quantitative and qualitative analyses will produce many informative and interesting results on their own. However, the main attraction of mixed methods and particularly of the convergent design is the possibility to fulfil the research equation 1 + 1 = 3 [37], where the knowledge gained is more than the sum of the individual quantitative and qualitative components. Figure 2 recapitulates the study flow.

To facilitate the emergence of this added value, we considered the pre-dialysis care trajectory as the same research object in both components that have definite and distinct objectives embedded in a complementarity oriented framework. After the completion of both quantitative and qualitative analyses, a series of questions can be asked and discussed. Do the overall results from both components converge or diverge concerning the reasons of ES? How can the risk factors of ES computed from the entire population be understood by the detailed speech of some patients and health professionals? To what extent and how do the types of pre-dialysis care trajectory from both components complete each other?

## 3. Discussion

This article presents a strategy to retrospectively study the care trajectories of patients with a chronic disease by harnessing the strength of both quantitative and qualitative research and using multiple data sources. Specifically, the aim of the mixed method described here is to gain a comprehensive understanding of the causes of emergency dialysis start of patients with CKD, an important public health issue. To our knowledge, this is the first study that will investigate the pre-dialysis care trajectory of patients with CKD using this methodology. The core of this methodology (i.e., studying specific variables of the trajectory in a large number of patients combined with the collection of depth and details regarding the trajectory, as a whole, of a few individuals) can be applied to other chronic diseases. For example, the trajectories of patients with chronic obstructive pulmonary disease could be retrospectively studied with our approach to identify facilitators of its early management for slowing down its progression and improving the overall care quality.

This design applied to the particular issue of dialysis start has strengths but also limitations that can be expected.

The main strengths of this approach include the use of individual data (clinical and healthcare consumption) at the national level for the entire population of interest, and the search for in-depth and crossed viewpoints of both patients and the main health professionals involved in CKD care. With the quantitative component, higher proportions of patients starting dialysis in emergency with particular characteristics and trajectories can be highlighted and risks factors can be identified. However, causality cannot be strictly established. Similarly, the qualitative analysis of extensive information from a limited number of patients and health professionals can add weight and robustness to the conclusions and the newly generated hypotheses, although it cannot establish causality. Another limit, inherent to this qualitative approach, is that data will not be collected for patients who do not agree to be interviewed or for whom language is a barrier. However, if applicable, their number and REIN registry characteristics will be described and discussed. Additionally, the retrospective nature of this research might introduce some bias. Particularly, reconstructing the patients’ trajectory is dependent on the patients’ views and how and what they choose to share. For example, patients might be prone to self-serving bias during the interview [38]. 

Integrating the two sets of results can be considered the main methodological challenge. It should be noted that data produced by registries and healthcare databases require a delay of consolidation (i.e., data on incident patients in 2015 were available only from 2018). Interviewing patients who started dialysis a few years before could introduce a major recall bias because some patients might not be able to recall precisely what happened at that time. In addition, and unfortunately, many dialyzed patients will not be alive any longer by the time their clinical/administrative data become available. For these reasons, the study population cannot be the same for the two components (i.e., patients who started dialysis in 2015 for the quantitative study, and incident patients in 2019 for the qualitative study). However, we do not think that the causes of emergency dialysis start or the patients’ characteristics will change during this short interval of time, and affect the integration process of the quantitative and qualitative results [15]. Nevertheless, it is possible that the integration process might reveal contradictions between result sets that could become the interest of additional investigations and provide new insights into the topic and generate new research questions.

Although the French national guidelines recommend initiating RRT preparation at least one year before its foreseeable start, we will assess two years of healthcare utilization data for our quantitative component, for a broader description of the trajectory. However, this study period should be considered along with the fact that the start of RRT of some CKD patients is rather unpredictable and their RRT preparation might have started several years before the actual dialysis start.

Results from this mixed method study will be published in scientific journals and presented at national and international conferences on nephrology and public health. Moreover, the identified barriers to a planned dialysis start and to the effective implementation of the existing clinical guidelines will be shared with the competent national health authorities to support the diffusion of co-management practices between GPs and nephrologists and the provision of high quality of care to non-dialyzed patients with CKD. 

## 4. Conclusions

This article describes an original mixed methodology to study the care trajectory of patients with ESRD and to identify the causes of an emergency start dialysis with robust and complementary findings. The described core methodology can be used and adapted to the study of other chronic diseases. 

## Figures and Tables

**Table 1:** Summary of the French Chronic Kidney Disease (CKD) care management guidelines [7].

Follow-Up Items	Stage 1, 2, and 3A (eGFR * ≥ 45)	Stage 3B (eGFR Between 30 and 44)	Stage 4 (eGFR Between 15 and 29)	Stage 5 (Before RRT) (eGFR < 15)
**Medical follow-up & consultation frequency**	**GP** once per year	**GP** at least once every 6 months **Nephrologist** at least once per year	**GP** at least once every 3 months **Nephrologist** at least once every 6 months	**GP** at least once per month **Nephrologist** at least once every 3 months
			RRT preparation, 1 year before its foreseeable start	
**Blood count**	-	Once every 6–12 months	Once every 1–3 months	Once every 1–3 months
**Serum creatinine**	Once per year	Once every 6 months	Once every 3–6 months	Once every 1–3 months
**Albuminuria**	Once per year	Once every 6 months	Once every 3 to 6 months	Nephrologist’s appreciation

* eGFR: estimated Glomerular Filtration Rate, expressed in mL/min/1.73 m²; RRT: renal replacement therapy; GP: general practitioner.

## Supplementary Materials

The following are available online at https://www.mdpi.com/1660-4601/16/24/5010/s1. Interview guide 1: Patient, Interview guide 2: General Practitioner, Interview Guide 3: Nephrologist.
