# Supplementary material for: The Pre-Dialysis Care Trajectory of Chronic Kidney Disease Patients and the Start of Dialysis in Emergency: A Mixed Method Study Protocol
Source: Int J Environ Res Public Health. 2019 Dec 9;16(24):5010. doi: 10.3390/ijerph16245010 (PMC6950758; doi:10.3390/ijerph16245010)
Supplement: Supplementary file 1 [file ijerph-16-05010-s001.pdf]

International Journal of
Environmental Research and Public Health

MDPI

Supplementary material 1: Patient Interview Guide

Patient presentation:

Could you introduce yourself?

1. First signs to CKD diagnosis

How and why are you here today?

1.1 First CKD signs and their interpretation

1) Can you tell me about how you first learned you had CKD?

3) Back then, what did you know about this disease?

4) Towards which healthcare professional have you been referred to and by who?

1.2 Diagnosis announcement

5) Can you tell me about the announcement of your diagnosis and your reaction?

7) What did the physician told you about it and what will follow?

8) What were the questions you had?

2. The pre-dialysis care and follow-up

2.1 Follow-up with the GP

10) What did your GP asked you to do regarding CKD?

11) How did the consultations with a GP take place? Could you describe a typical or an atypical one?

13) What do you think about the follow-up you had with a GP?

2.2 Follow-up with the nephrologist

14) How did the consultations with a GP take place?

15) Did you experience any difficulty to see him?

16) What information were you given regarding CKD care?

17) How did you discuss the next steps of your care with the nephrologist, specifically renal replacement therapy?

18) What do you think about the follow-up you had with a nephrologist?

2.3 Other healthcare professionals

19) Before CKD, did you have any particular health problems?

20) What other health professionals did you encounter?

21) Could you tell me more about the different treatment you had before dialysis?

22) Have you participated in activities aimed at managing a disease or live with it?

3. Transition from CKD to ESRD and dialysis

3.1 Dialysis preparation

23) How did you learn about dialysis for the first time?

24) Before starting dialysis, what did you know about it?

25) Were you prepared for dialysis? How did that preparation go?

3.2 Emergency start dialysis

26) Could you tell me about your first dialysis and how did it go?

27) Before this first dialysis, when was the last time you saw your nephrologist?

4. Living with and managing CKD

Before dialysis, how did you live with CKD?

4.1 Living with CKD and its consequences

28) Before dialysis, what did CKD changed in your life?

29) When you had a question or difficulty regarding CKD, what did you do?

30) Did you or are you part of a patient advocacy group or any support group?

4.2 Perspectives

31) How are your dialysis sessions going now?

32) How do you see the future?

Supplementary material 2: General Practitioner (GP) Interview Guide

GP's presentation and activity

How long have you been practicing medicine?

What geographical area do you cover?

How much of your activity covers chronic kidney disease (CKD)?

Could you tell me about a trajectory of one of those patients?

1. GP's practice

1.1. Screening and diagnosis

1) How does the screening and diagnosis of CKD takes place?

2) How do you assess patients' need regarding CKD?

3) How does the CKD diagnosis announcement takes place?

1.2. Follow-up

4) What are the specificities of a consultation with a CKD patient?

5) What do you think are the role of the GP in the CKD follow-up?

6) What is behind the therapeutic education regarding CKD patients?

7) What are the drug and non-drug treatments that you offer to CKD patients?

8) Today, do you feel at ease with CKD?

1.3. Collaboration

9) When and in what situations do you refer a patient to a nephrologist?

10) How does the collaboration with a nephrologist translate in your practice?

11) What do you do in case of CKD complications?

2. Knowledge and views regarding guidelines

2.1. Knowledge

12) During your studies, did you have any CKD specific courses?

13) National guidelines regarding CKD management have been published in 2012, what are your thoughts on it?

2.2. Implementation

14) More generally, what do you think of the clinical guidelines you receive?

15) What place do they occupy in your daily practice?

3. Transition from CKD to End Stage Renal Disease (ESRD)

16) When CKD reach its last stage, how is decided what happens next regarding patients' care?

17) What do you think of dialysis as a treatment?

18) How is discussed the need to start dialysis with your patients?

19) Based on your experience, how do you think patients approach the start of dialysis?

20) What do you do when faced with a patient's reluctance to start dialysis?

21) What does an emergency dialysis mean to you?

Supplementary material 3: Nephrologist Interview Guide

Nephrologist's presentation and activity

How long have you been practicing medicine?

What brought you to specialize in nephrology?

What geographical area do you cover?

How many patients do you follow?

What are the specificities of CKD?

Guidelines regarding the care trajectory of CKD have been published in 2012 what does « care trajectory » mean to you?

1. Nephrologist's practice

1.1. Follow-up

1) How does the first consultation with a CKD patient takes place?

2) Could you tell me about a trajectory of one of those patient?

3) What does the follow-up consist of, at your level?

4) What is behind the therapeutic education regarding CKD patients?

5) What are the drug and non-drug treatments that you offer to CKD patients?

6) What are deterioration factors that you look for?

1.2. Collaboration

7) CKD management guidelines talk about « shared co-management » with other health professionals, what does it mean to you? With the GP?

8) What do you think the role of a GP is regarding CKD?

2. Knowledge and views regarding guidelines

2.1. Knowledge

9) What do you think of the 2012 CKD management guidelines?

10) How do you stay up to date regarding your professional practices, particularly CKD follow-up and dialysis start?

2.2. Implementation

11) More generally, what do you think of the clinical guidelines you receive?

12) What place do they occupy in your daily practice?

3. Transition from CKD to End Stage Renal Disease (ESRD)

13) How is the decision to start dialysis taken?

14) How do you choose one treatment among the others?

15) How is discussed the need to start dialysis with your patients?

16) What is offered to the patient regarding dialysis preparation?

17) What do you do when faced with a patient's reluctance to start dialysis?

18) What does an emergency dialysis mean to you?

19) Some patients start dialysis in emergency, what do you make out of it?
